# Prevention of preterm delivery by cervical cerclage; a comparison of prophylactic and emergency procedures

**DOI:** 10.4274/jtgga.galenos.2020.2019.0183

**Published:** 2021-02-24

**Authors:** Seda Yüksel Şimşek, Erhan Şimşek, Gülşen Doğan Durdağ, Songül Alemdaroğlu, Şafak Yılmaz Baran, Hakan Kalaycı

**Affiliations:** 1 Clinic of Obstetrics and Gynecology, Başkent University Adana Dr. Turgut Noyan Application and Research Hospital, Ankara, Turkey

**Keywords:** Cervical insufficiency, cervical cerclage, preterm birth, neutrophil-lymphocyte ratio

## Abstract

**Objective:**

Prophylactic or emergency type cervical cerclage procedures are being used for treatment of cervical insufficiency. The aim was to review and compare the outcomes of these cerclage types and identify factors affecting outcomes.

**Material and Methods:**

Retrospective review of seventy-five patients in whom transvaginal cervical cerclage procedures were performed over a seven-year period in a tertiary referral center.

**Results:**

Twenty seven of 75 (36%) patients were in the emergency cerclage group and 48 (64%) of them were in the prophylactic group. Mean body mass index (BMI), hospitalization time and gestational week at cerclage were significantly higher, whereas latency period was significantly shorter for the emergency group. Mean gestational ages at delivery were 35.6±4.5 and 33.6±5.9 weeks in the prophylactic and emergency groups, respectively (p=0.117). Delivery rates under 34^th^ gestational week were 20.8% and 37.0% in the prophylactic and emergency groups, respectively (p=0.175). Birthweight, and delivery ≥34^th^ gestational week was higher in the prophylactic group, whereas complication rate was higher in the emergency group, but these differences were not significant. High BMI was associated with more deliveries before 34-week in the prophylactic group. Pre-cerclage cervical length was shorter in patients who delivered before 34 gestational weeks at delivery.

**Conclusion:**

Prophylactic and emergency cerclage procedures have comparable results regarding gestational week at delivery. High BMI and low pre-cerclage cervical length may have adverse effects on success of cerclage procedures.

## Introduction

Cervical insufficiency can be described as an inability of cervix uteri to retain the pregnancy in the absence of objective signs of labor, for example due to normal uterine contraction, especially in the second trimester. It has a particular clinical importance since preterm birth and prematurity-related risks are high in this group of patients. The incidence is reported to be around 1% in the general obstetric population, but this rate is 8% in women with second trimester pregnancy loss (1). The etiology of cervical insufficiency is not clear but risk factors include antecedent cervical surgeries such as conization, repeated dilatation and curretage, congenital uterine anomalies, in utero exposure to the synthetic estrogen, diethylstilbestrol and, possibly, the most important risk factor is a history of cervical insufficiency in previous pregnancies (2). Bed rest, activity restriction and vaginal pessaries are non-surgical treatment modalities for cervical insufficiency and the effectiveness of these modalities has been evaluated previously (3,4,5). Activity restriction was reported to be ineffective in one study (6). Moreover, a higher risk of preterm delivery has been reported in women advised to restrict activity. In singleton pregnancies diagnosed with short cervix, expectant management was compared with vaginal pessaries and pessaries were shown to be more effective at reducing delivery under 34 gestational weeks. However, in twin pregnancies, vaginal pessary was not superior to expectant management in preventing delivery under 34 gestational weeks in a contemporary publication. Due to a lack of consensus in identifying the optimal non-surgical treatment, these modalities are generally discouraged (2).

Cervical cerclage procedures can be performed transabdominally or transvaginally. Transabdominal approaches should be reserved for patients with cervical anatomical disturbances, such as trachelectomized patients, and also for patients with repetitive failure of transvaginal cerclage that resulted in pregnancy loss. McDonald- and Schirodkar-type transvaginal cervical cerclage are the best known and most widely performed and both are equally effective (7).

Indication for cerclage can be based on medical history or as a result of findings uncovered during physical examination often requiring emergency cerclage procedures. The American College of Obstetrician and Gynecologists (ACOG) define indications for prophylactic cerclage as painless cervical dilatation or a requirement for cervical cerclage in a prior pregnancy. ACOG guidelines recommend that indications for emergency cerclage include painless cervical dilatation in the second trimester and cervical length under 25 mm with a history of preterm birth before 34 gestational weeks in a prior pregnancy (2).

Success of these cerclage procedures in preventing preterm delivery may be affected by a range of clinical parameters and patient characteristics. The aim of this study was to analyze and compare outcomes of prophylactic and emergency cerclage performed in a tertiary referral center. A further aim was to delineate factors that can affect the efficiency of cervical cerclage which may include body mass index (BMI), pre-cerclage cervical length and neutrophil-lymphocyte ratio.

## Material and Methods

### Patients

Cervical cerclage procedures performed between January 2012 and February 2019 were reviewed retrospectively from hospital records. Pregnancy and labor information were obtained by telephone-based questioning, when hospital records were incomplete. ACOG recommendations on cerclage indications were taken as guidelines. Prophylactic or history based cerclage was applied between 11-14 gestational weeks, after first trimester screening tests, for patients with history of cervical insufficiency in previous pregnancy. Emergency cerclage was performed for patients with painless cervical dilatation in the second trimester and also for patients with preterm birth history and diagnosis of short cervix in the current pregnancy. Cervical cerclage procedures were not performed in the presence of regular uterine contractions, active vaginal bleeding, chorioamnionitis, fetal anomaly, rupture of membranes and dilated cervix beyond 3 cm.

### Ethics

This study was approved by the Başkent University Faculty of Medicine Institutional Ethical Committee on 05/14/2019, with the approval number of KA19/168. Informed consent of patients was obtained before cervical cerclage procedures.

### Interventions

Pre-cerclage cervical length was measured by transvaginal ultrasound (TVUS), with empty bladder. At least three measurements were taken and the mean value was calculated. The degree of cervical dilatation was also measured by TVUS, under sterile conditions. McDonald cervical cerclage was applied to all patients and Schirodkar type cerclage was not used in this study population. Sterilization was achieved by the application of povidone iodine to the vagina and cervix under sedoanalgesia. The anterior portion of the cervix was grasped with an oval clamp and sutured at the twelve, nine, six and three o’clock positions. Non-absorbable braided suture material was used for this (Cervix-set B. Braun Surgical S.A.). Prolapsed membranes were relocated by placing and inflating a pediatric Foley catheter into the cervical canal in those patients with a dilated cervix. Prophylactic antibiotic, intramuscular progesterone and indomethacine were given to all patients, postoperatively. Postoperative complications were defined as massive vaginal bleeding, chorioamnionitis and premature rupture of membranes. Hospitalization time was defined as the period from operation until discharge.

### Statistical analysis

Statistical analysis was performed using SPSS, version 17.0 (IBM Inc., Chicago, IL, USA). If continuous variables distributed normally, they are described as mean ± standard deviation (p>0.05 in Kolmogorov-Smirnov test or Shapiro-Wilk n<30) and if continuous variables did not distribute normally, they are described as median (range). Continuous variables were compared using Student’s t test when normally distributed and using Mann-Whitney U test when did not distribute normally. Categorical variables were compared between groups by chi-square or Fisher’s exact test. Values of p<0.05 were considered to indicate statistical significance.

## Results

**Figure 1:**
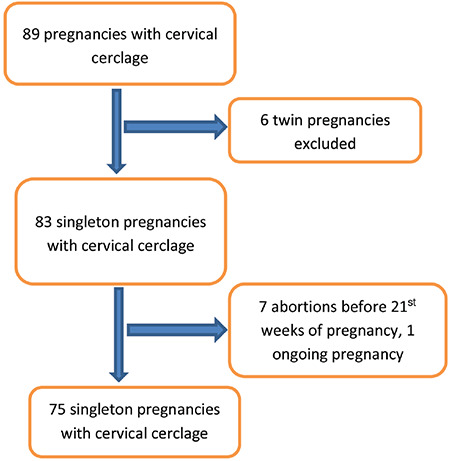
Flow diagram of the study

**Figure 2:**
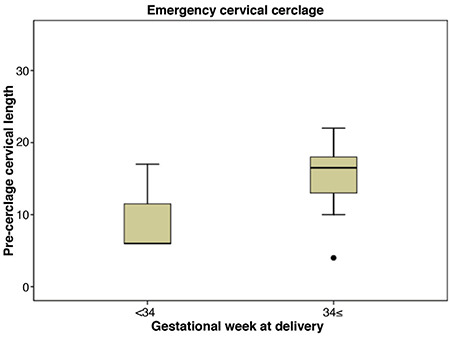
Pre-cerclage cervical length and gestational week at delivery in the emergency cerclage group

**Figure 3:**
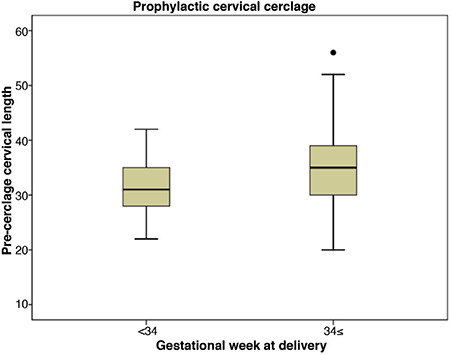
Pre-cerclage cervical length and gestational week at delivery in the prophylactic cerclage group

During the study period, a total of 89 cervical cerclage procedures were performed in this tertiary center. Twin pregnancies and pregnancies that ended before 21^st^ week of gestation were excluded from the study (Figure 1). As a result, 75 patients with singleton pregnancies and diagnosis of cervical insufficiency were included in the study. Twenty-seven of 75 patients were in the emergency cerclage group and 48 were in the prophylactic group. Mean BMI value, hospitalization time and gestational week of cerclage application were significantly higher in the emergency group compared to prophylactic group. Latency period, which is from cerclage week to delivery week was significantly shorter for the emergency cerclage patients (14.2±6.5 vs 21.7±4.8 weeks; p<0.001). Nonetheless, there were no statistically significant differences between the two groups regarding other clinical and demographic parameters (Table 1). The effect of BMI, preoperative cervical length and neutrophil-lymphocyte ratio on the week of delivery under and above 34 gestational weeks were evaluated. In the prophylactic cerclage group, patients who gave birth before 34 gestational weeks had significantly higher BMI values than the values of those giving birth after 34 gestational weeks (28.2±4.4 vs 25.0±4.2; p=0.04). There was a similar trend in the emergency cerclage group but the difference was not significant (31.8±10.6 vs 28.0±3.4; p=0.186) (Table 2). In the prophylactic group, mean pre-cerclage cervical length of patients who delivered before and after at 34 gestational weeks was not different; 30.9±5.3 mm vs 35.1±7.9 mm (p=0.117) respectively. In the emergency group these values were 9.6±6.3 mm vs 16.6±6.7 mm (p=0.136), respectively (Figure 2, 3).

In the emergency cerclage group, in patients with dilated cervix, the proportion giving birth before 34 weeks was 70%. This proportion dropped to 30% in the group with a diagnosis of short cervix only in the absence of cervical dialtation. However, the difference was still not significant (p=0.120; Table 3).

## Discussion

The main determinant of success for a cervical cerclage procedure is the capability of preventing preterm birth and related adverse outcomes. In this cohort the prophylactic cerclage group had a higher mean gestational age, higher birth rate above 34 gestational weeks, higher mean gestational weight, and lower complication rate than the emergency cerclage group. However, the mean gestational week at delivery did not differ between the two groups.

Gestational weeks completed and perinatal outcome have been compared previously in history-based and ultrasound-based cervical cerclage patients (8,9). Gluck et al. (10) compared the obstetric outcomes of patients admitted with cervical dilatation or shortened cervical length to history-indicated cerclage patients and gestational week at delivery and birthweights were similar for both groups. Liddiard et al. (11) also did not find any significant difference in gestational week, birthweight, live birth rate or requirement for neonatal intensive care unit (NICU) between emergency and prophylactic cerclage groups (11). In the same study, the complication rate in emergency cerclage patients was higher than in the prophylactic cerclage group, but it should be noted that approximately half of the patients in the emergency group had twin pregnancies and at least 3 cm cervical dilatation at admittance. In a recently published meta-analysis, which also included the Gluck et al. (10) and Liddiard et al. (11) studies, birth week and birth weights were found to be significantly lower and the risk of membrane rupture higher in the emergency cerclage group (12). In our cohort, age and pregnancy types were similar in both groups, but BMI values were significantly higher amongst emergency cerclage patients (p=0.006). Mean gestational week at delivery and mean birth weight tended to be higher in the prophylactic cerclage group, whereas complication rate and delivery under 34 gestational weeks tended to be greater in the emergency cerclage group. Only hospitalization time was significantly different between the prophylactic and emergency groups.

In emergency cases, there is a process that has already started and is ongoing; short or dilated cervix was recognized as the sign of an impending threat of cervical insufficiency. In prophylactic cases, however, there is usually a well-known history of cervical insufficiency in a previous pregnancy, so both patient and physician are well-prepared for clinical situations and required treatment options in an on-going pregnancy. It is reasonable to assume that forestalling a process that has not yet started is clinically easier than forestalling one that has already started. The differences observed between the two groups in our study may be partially explained this way. Nevertheless, since differences were not significant regarding delivery week, birthweight, complication rate, and requirement for NICU admission, we can conclude that outcomes of both cerclage types are similar and comparable. Latency period from cerclage to delivery was significantly higher in the prophylactic group, but gestational week at cerclage was higher in the emergency group, as expected. Since the mean cerclage week was significantly earlier (13.9 weeks) in the prophylactic group compared with 19.4 in the emergency group the longer latency period of the prophylactic group can be partially attributed to this difference.

A cervical cerclage procedure is recommended, with evidence level IA, for patients who had spontaneous preterm birth or had been diagnosed cervical insufficiency in previous pregnancies and have a cervical length under 25 mm in their current pregnancy (1). Berghella and Mackeen (13) published a meta-analysis including four randomized controlled trials and concluded that patients with a history of cervical insufficiency can be safely followed by serial TVUS cervical length measurement. This study concluded that cerclage procedures as a result of medical history may be unnecessary and may be reduced. A retrospective study of Brown et al. (8) found approximately 50% of patients with history did not require cerclage when followed by serial TVUS measurements. Moreover, the obstetrical and perinatal outcomes were similar between history-based and ultrasound-based cerclage groups. The main aim of serial cervical length measurements in patients with a history of cervical insufficiency is to reduce unnecessary cerclage procedures and related complications. In this study, ACOG’s criteria were followed and cervical cerclage performed between 11-14 weeks of pregnancy in patients with a history of cervical insufficiency in a previous pregnancy. Cervical lengths were measured just before the procedure by TVUS. Mean cervical length of patients delivered at and after 34 gestational weeks did not differ from that of deliveries under 34 gestational week in the prophylactic cerclage group. There was also no difference in mean cervical length in the emergency cerclage group when comparing deliveries before and after 34 gestational weeks. As a result, an increase in pre-cerclage cervical length was associated with improvement of gestational week at delivery, although differences were not significant in this study. In patients requiring emergency cerclage, those who were admitted with cervical dilatation had a greater proportion within deliveries before 34 gestational weeks compared to the emergency cerclage patients without cervical dilatation, although this was again not significant. We suggest that the small sample sizes may have made our findings unreliable, otherwise it is highly probable that improvement of gestational week at delivery is directly proportional with pre-cerclage cervical length.

High BMI has been associated with various adverse pregnancy and obstetric outcomes. The effect of BMI on cerclage procedures has also been studied. Suhag et al. (14) investigated the effect of pre-pregnancy BMI on the success of history-indicated and ultrasound-indicated cerclage and reported no effect of BMI. In another retrospective observational study, no association was found between BMI and latency period (15). Interestingly, Schirodkar-type cerclage was reported to be superior to McDonald-type in obese patient groups in terms of better gestational week at delivery (16). One study showed an inverse proportion between BMI and gestational week at delivery in history-indicated cerclage patients (17). In the present study the mean BMI was significantly greater in the emergency cerclage group compared to the prophylactic cerclage patients. This difference may be due to the general adverse effect of higher BMI on obstetrical outcomes. There was a significant inverse relationship between gestational week at delivery and BMI in the prophylactic cerclage patients. A similar, but non-significant, trend was observed in the emergency cerclage patients. In general, high BMI values appeared to have a negative effect on cerclage efficiency. This should be taken into account when counseling patients, pre-procedurally.

The neutrophil-lymphocyte ratio is accepted as an indicator of the presence of pro-inflammatory processes. The prognostic value of this parameter has been investigated in chronic and acute inflammatory conditions and oncologic disease (18,19). The utility of neutrophil-lymphocyte ratio has been investigated in obstetric conditions including ovarian torsion and preeclampsia (20,21). Since delivery is a pro-inflammatory process, one can speculate that preterm delivery is also such a process and the neutrophil-lymphocyte ratio may be useful in predicting early delivery. A relationship has been reported between increase in neutrophil-lymphocyte ratio and delivery under 28 gestational weeks in patients with recurrent cervical cerclages (22). In our study, there was no difference in neutrophil-lymphocyte ratio in the emergency and prophylactic cerclage patients (p=0.196). In a subgroup analysis, neutrophil-lymphocyte ratio in emergency cerclage patients was higher, but not significantly so, in the group delivering after 34 gestational weeks. Similarly, in the history-indicated prophylactic cerclage patients, neutrophil-lymphopcyte ratio was non-significantly higher in patients who delivered at and after 34 gestational weeks. There appears to be a tend towards higher neutrophil-lymphocyte ratio in deliveries after 34 gestational weeks, but no significance was found and in addition, group sizes were small, so no reliable conclusion can be drawn.

### Study Limitation

The main limitation of this study is its retrospective nature. Furthermore, results are robust, since strict criteria were applied in order to identify patients who were candidates for prophylactic and emergency cerclage procedures. Also, number of included patients is not that insufficient when we consider the incidence of cervical incompetence but the small group, and in particular the small sub-group analyses make statistical comparisons less reliable.

## Conclusion

Main indications for prophylactic cervical cerclage procedures are well defined in contemporary and evidence-based guidelines. The results of the present study indicated that emergency cerclage procedures may improve gestational week at delivery. Similarly; to prophylactic cerclage procedures, emergency cerclage procedures may be effective in preventing preterm and severely preterm deliveries. This result should not be interpreted as emergency cerclage being as effective as prophylactic cerclage. Emergency cerclage cannot substitute for a prophylactic procedure. Liberal use of cerclage may have adverse outcomes and risks, but when used with appropriate indication, the procedure improve obstetrical and perinatal outcomes. Pre-cerclage cervical length correlated with gestational week at delivery in both prophylactic and emergency cerclage groups. Cervical dilatation at admission may be a poor prognostic factor for preterm delivery in emergency cerclage patients. In all cerclage patients higher BMI values have a negative effect on gestational week at delivery. In addition, the prognostic value of neutrophil-lymphocyte ratio remains unclear. Larger randomized studies may illuminate the relationship between these factors and cerclage procedure outcomes.

## Figures and Tables

**Table 1:**
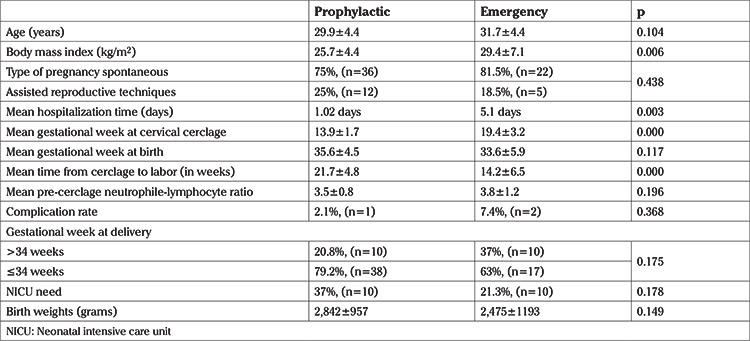
Characteristics and perinatal outcomes of patients in emergency and prophylactic cervical cerclage patients

**Table 2:**
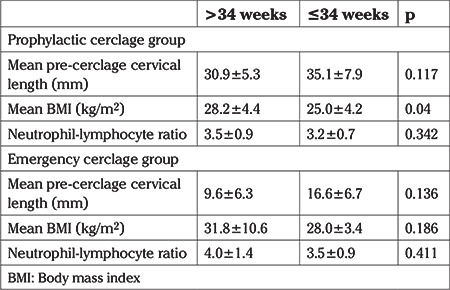
Pre-cerclage cervical length, BMI, and neutrophil-lymphocyte ratio by gestational age at delivery

**Table 3:**
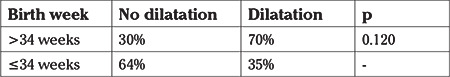
Gestational week at delivery according to presence or absence of cervical dilatation, in emergency cerclage patients
